# CD44 Variant Expression in Follicular Cell-Derived Thyroid Cancers: Implications for Overcoming Multidrug Resistance

**DOI:** 10.3390/molecules30091899

**Published:** 2025-04-24

**Authors:** Benny Mosoane, Michelle McCabe, Brandon S. Jackson, Zodwa Dlamini

**Affiliations:** 1 Department of Anatomical Pathology, Faculty of Health Sciences, University of Pretoria, Pretoria 0001, South Africa; michelle.mccabe@nhls.ac.za; 2 Breast and Endocrine Unit, Department of General Surgery, University of Pretoria, Kalafong Provincial Tertiary Hospital, Pretoria 0001, South Africa; brandon.jackson@up.ac.za; 3 SAMRC Precision Oncology Research Unit (PORU), DSI/NRF SARChI Chair in Precision Oncology and Cancer Prevention (POCP), Pan African Cancer Research Institute (PACRI), University of Pretoria, Pretoria 0001, South Africa

**Keywords:** CD44 variants, thyroid cancer, multidrug resistance, follicular cell-derived carcinoma, therapeutic targets, tumor microenvironment

## Abstract

Thyroid cancer (TC) is a significant global health issue that exhibits notable heterogeneity in incidence and outcomes. In low-resource settings such as Africa, delayed diagnosis and limited healthcare access exacerbate mortality rates. Among follicular cell-derived thyroid cancers—including papillary (PTC), follicular (FTC), anaplastic (ATC), and poorly differentiated (PDTC) subtypes—the role of CD44 variants has emerged as a critical factor influencing tumor progression and multidrug resistance (MDR). CD44, a transmembrane glycoprotein, and its splice variants (CD44v) mediate cell adhesion, migration, and survival, contributing to cancer stem cell (CSC) maintenance and therapy resistance. Differential expression patterns of CD44 isoforms across TC subtypes have shown diagnostic, prognostic, and therapeutic implications. Specifically, CD44v6 expression in PTC has been correlated with metastasis and aggressive tumor behavior, while in FTC, its expression aids in distinguishing malignant from benign lesions. Furthermore, CD44 contributes to MDR through enhanced drug efflux via ABC transporters, apoptosis evasion, and CSC maintenance via the Wnt/β-catenin and PI3K/Akt pathways. Targeted therapies against CD44 such as monoclonal antibodies, hyaluronic acid-based nanocarriers, and gene-editing technologies hold promise in overcoming MDR. However, despite the mounting evidence supporting CD44-targeted strategies in various cancers, research on this therapeutic potential in TC remains limited. This review synthesizes existing knowledge on CD44 variant expression in follicular cell-derived thyroid cancers and highlights potential therapeutic strategies to mitigate MDR, particularly in high-burden regions, thereby improving patient outcomes and survival.

## 1. Introduction

Thyroid cancer (TC) represents a significant public health concern worldwide, with its incidence and mortality rates demonstrating notable geographic variation. The most common types, papillary thyroid carcinoma (PTC) and follicular thyroid carcinoma (FTC), are classified as differentiated thyroid cancers (DTCs) and originate from the epithelial cells of the thyroid follicles through a cascade of genomic dysregulation [1]. FTC, often associated with iodine deficiency, shows considerable prevalence in iodine-deficient regions like West Africa, where FTC comprises up to 35% of thyroid malignancies [2]. In South Africa, despite the introduction of ionized table salt in 1982, FTC exhibits more prevalence than expected [3]. In contrast, anaplastic thyroid carcinoma (ATC), a highly aggressive form of TC, also exhibits a higher prevalence in regions with endemic FTC. Another subtype, poorly differentiated thyroid carcinoma (PDTC), represents a rare but clinically significant form of TC, displaying intermediate aggressiveness between DTC and ATC. Late-stage presentations and limited treatment options for ATC and PDTC contribute to poor survival outcomes [4,5].

TC poses a substantial clinical burden in low- and middle-income countries, particularly in Africa. Mortality rates are disproportionately high, influenced by factors such as delayed diagnosis, limited access to healthcare, and regional variations in iodine deficiency [6,7]. The aggressive nature of certain TC subtypes, such as ATC and PDTC, and the higher prevalence of FTC in iodine-deficient areas compound these challenges. Despite advancements in surgery, radioiodine therapy, and targeted treatments, a subset of patients with TC develops resistance to conventional therapies, leading to poor outcomes. In advanced and recurrent differentiated thyroid tumors, chemoresistance is more prevalent, a result that is characterized by significant molecular variability in both spatial and intra-tumoral heterogeneity [8]. This heterogeneity, driven by genetic and epigenetic alterations, contributes to the emergence of multidrug resistance (MDR), a critical barrier in TC [9,10]. Addressing MDR requires a deeper understanding of the molecular mechanisms underlying resistance, particularly those involving cancer stem cells (CSCs) and their associated markers, such as cluster of differentiation 44 (CD44) variants.

CD44 is a family of transmembrane glycoproteins with diverse roles in cell adhesion, migration, and signal transduction. CD44 and its variants, generated through alternative splicing, have been implicated in various human malignancies, including TC. These variants, particularly CD44v6 and CD44v8-10, have been implicated in TC progression, metastasis, and chemoresistance [11]. The expression of CD44v6 in PTC has been associated with lymph node involvement, suggesting its role in tumor metastasis. In ATC, CD44v6 is markedly overexpressed and contributes to tumor development and fostering an aggressive phenotype. Similarly, CD44v8-10 expression in PTC is correlated with tumor aggressiveness and poor prognosis [12].

Despite their role in tumor classification, CD44 variants play a critical role in maintaining CSC populations, which are inherently resistant to chemotherapy and radiation. The self-renewal capacity of CSCs and their ability to evade apoptosis complicates the treatment outcomes in TC [13,14,15,16]. CD44-mediated activation of signaling pathways such as Wnt/β-catenin and PI3K/AKT further promote tumor survival and MDR, reinforcing CD44s as key therapeutic targets in TC [17]. Furthermore, the diagnostic potential of CD44 isoforms extends beyond PTC and ATC. In FTC, CD44v6 expression varies significantly from that in follicular adenoma (FA), enabling a distinction between benign and malignant lesions [18]. This differential expression highlights the biomarker potential of CD44 isoforms in refining TC diagnosis.

Given the role of CD44 in promoting tumor resistance, targeting CD44 and its associated pathways has emerged as a promising strategy for overcoming MDR. Studies exploring the combination of non-steroidal anti-inflammatory drugs with heat shock protein 90 inhibitors have demonstrated potential in overcoming MDR by targeting CD44-overexpressing CSCs [19,20].

This review aims to consolidate current knowledge on the expression of CD44 variants in follicular cell-derived thyroid cancers, with a focus on their implications for MDR. By exploring the molecular mechanisms of CD44-mediated drug resistance and its potential as a therapeutic target, this review seeks to highlight pathways for advancing the management of TC, particularly in regions with high disease burden.

## 2. CD44 Variants: Structure, Isoforms, and Functions

### 2.1. Molecular Structure of CD44

**Figure 1:**
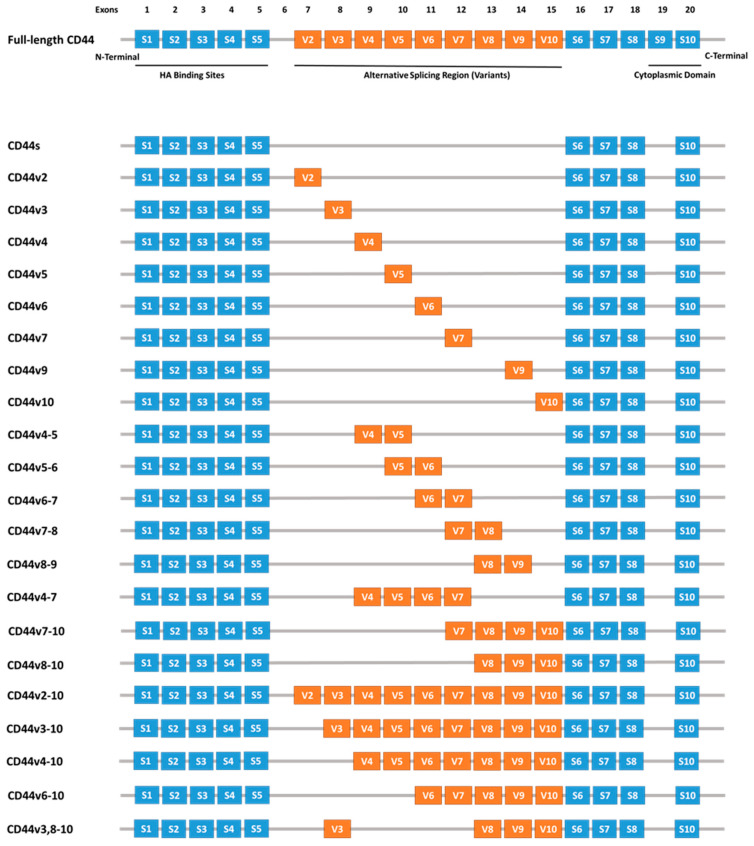
Schematic representation of the human CD44 gene, highlighting constitutive exons (blue boxes) and variable exons (orange boxes) produced through alternative splicing. Abbreviations: CD44—Cluster of Differentiation 44, S—Standard, V—variant [23].

CD44 is a cell surface glycoprotein encoded by the CD44 gene located on chromosome 11p13 [21]. This transmembrane protein exhibits diverse structural and functional properties, playing critical roles in various physiological and pathological processes. The transcription of the CD44 gene involves alternative splicing, resulting in two main isoforms: the standard isoform (CD44s) and the variant isoform (CD44v). In humans, the CD44 gene comprises 19 exons, with exons 1–5 and 16–20 being constitutive. The remaining exons (6–15) undergo alternative splicing to produce CD44v, Figure 1 [22].

CD44 expression is also regulated by post-transcriptional modifications, including alternative splicing and mRNA processing [24]. These processes contribute to the functional and structural diversity of the protein, enabling its role in regulating cell adhesion, migration, and signaling. Abnormal splicing and dysregulation of CD44 isoforms have been linked to tumor progression and resistance to therapy, highlighting their significance in cancer biology [25,26].

**Figure 2:**
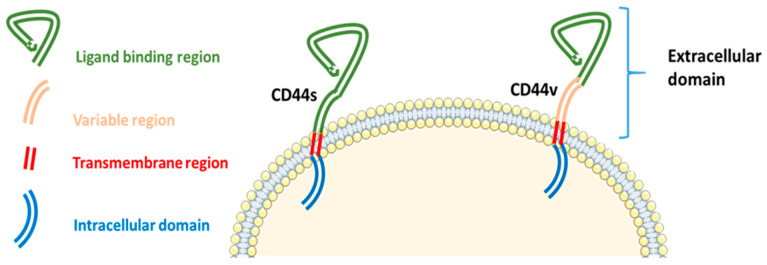
Structure of the CD44 protein, illustrating its extracellular, transmembrane, and intracellular domains with the localization of variant isoforms. CD44—Cluster of Differentiation 44, S—Standard, V—variant [23].

Structurally, CD44 consists of three domains: an extracellular domain, a transmembrane domain, and an intracellular domain. Variants of CD44 are localized within the extracellular domain, which mediates interactions with the extracellular matrix (Figure 2) [23]. The highly conserved transmembrane and intracellular domains are essential for membrane localization, ligand binding, and the subsequent downstream intracellular events. These domains interact with cytoskeletal elements and signaling molecules, facilitating the transmission of extracellular signals to intracellular pathways that regulate cellular processes such as proliferation, migration, and adhesion [27].

### 2.2. Physiological Roles of CD44

CD44 is widely expressed across human tissues, with significant roles in organ differentiation, lymphocyte homing, leukocyte activation, lymphopoiesis, cytokine release, cell adhesion, migration, and angiogenesis [28,29,30]. Furthermore, CD44 expression is detected in embryonic tissues, including oocytes, early embryos, pre-hatched blastocysts, and placental stromal cells, suggesting its importance in early development [31,32,33].

The CD44 isoform is ubiquitously expressed in tissues, including the skin, lungs, liver, pancreas, oral cavity, esophagus, and central nervous system [29]. In contrast, CD44v isoforms exhibit restricted expression, predominantly found in activated lymphocytes, macrophages, and epithelial cells of certain tissues, such as the uterine cervix, bladder, and stomach [28,34]. For instance, CD44v6 expression is confined to the basal keratinocytes of cervical squamous epithelium [35,36].

CD44 interacts with several ligands, including hyaluronic acid (HA), serglycin, and osteopontin, playing essential roles in regulating physiological processes such as cell adhesion, migration, extracellular matrix (ECM) remodeling, and maintaining tissue homeostasis [37,38]. These interactions support the dynamic balance necessary for normal cellular function and structural integrity. The extracellular domain of CD44 engages with these ligands to activate signaling pathways that regulate cell proliferation and migration [37]. Additionally, the interaction between CD44 and matrix metalloproteinases (MMPs) contributes to ECM homeostasis and cell signaling, further highlighting its role in physiological regulation [39,40,41].

### 2.3. CD44 Expression Regulation in Cancer

CD44 expression in cancer is regulated by multiple mechanisms, including transcriptional, post-transcriptional, and epigenetic changes. The transcription factors NF-κB, AP-1, and HIF are key regulators of CD44 upregulation in response to various environmental stimuli, including inflammation, hypoxia, and stress. MicroRNAs, including miR-34a and the miR-200 family, also modulate CD44 expression by targeting its mRNA, influencing processes such as epithelial-mesenchymal transition (EMT) and tumor growth [42]. Furthermore, alternative splicing of CD44, regulated by splicing factors such as SR proteins, generates CD44s and CD44v isoforms, wherein the latter is often associated with tumor metastasis and chemoresistance. These splicing events contribute to enhancing the aggressive behavior of cancer cells, particularly in CSCs [43].

Apart from transcriptional and splicing control, post-translational modifications such as glycosylation and phosphorylation also regulate CD44’s functional activity, modulating its interaction with ECM components and other cell surface receptors [44,45]. The growth factor- and cytokine-rich tumor microenvironment also significantly contributes to CD44 regulation. Transforming growth factor-β, epidermal growth factor, and fibroblast growth factor are factors that activate signaling pathways to upregulate CD44, promoting tumor cell survival, invasion, and resistance to chemotherapy [13,44,46].

### 2.4. CD44 Variants in Cancer

Notably, CD44 isoforms are highly expressed in certain differentiated carcinomas, whereas minimal expression is observed in tumors such as melanoma, neuroblastoma, and Burkitt lymphoma [47,48,49]. HA binds to the N-terminus of CD44 isoforms, influencing physiological and pathological processes [50]. Iron-mediated endocytosis of the HA-CD44 complex is critical for cancer progression, invasion, and chemoresistance [51]. Clinical studies suggest patients with CD44s-positive tumors may benefit from HA–irinotecan, while HA-based nanocarriers in CD44-overexpressing tumors enhance drug delivery, increase therapeutic efficacy with low cytotoxicity, inhibit tumor growth, and enable targeted chemotherapy [37,52].

Osteopontin modulates proliferation, invasion, and inflammation via signaling pathways, such as JUN N-terminal kinase activation, promoting tumor growth in xenograft models [53]. Serglycin, another CD44 ligand, facilitates tumorigenesis through its glycosaminoglycans-mediated binding, regulating CD44 expression via β-catenin signaling and promoting migration in non-small cell lung cancer [54,55]. In giant cell tumors of bone, serglycin–CD44 interactions activate focal adhesion kinase, driving tumor progression [56]. In malignancy, CD44 interacts with MMPs and fibronectin, influencing ECM dynamics and cellular processes such as adhesion, migration, and invasion [37]. Dysregulation in CD44–MMP and CD44–fibronectin pathways contributes to pathological conditions, highlighting their potential as therapeutic targets for restoring physiological balance in cancer [57].

Differential expression of CD44 variants has been implicated in tumorigenesis and metastasis [23,58]. In a study by Resnick et al., CD44 expression was examined in primary and metastatic tumors, as well as cell lines derived from CNS tumors. CD44v-positive breast cancer cell lines showed strong expression compared to weak CD44v in primary brain tumors and normal brain tissues. However, metastatic brain tumors exhibited high levels of isoforms encoding multiple variant exons, while spinal metastatic tumors showed minimal CD44v expression, suggesting CD44v’s role in influencing metastatic site selection within the CNS [59].

In PTC, the CD44v8-10/CD44s mRNA expression ratio was found to increase significantly with age. Additionally, this ratio showed a notable negative correlation with cell proliferation [12]. These findings suggest that the CD44v8-10/CD44s expression ratio could serve as a useful marker for identifying aggressive PTC, offering valuable clinical insights.

## 3. CD44 Variant Expression in Follicular Cell-Derived Thyroid Cancers

A handful of studies have focused on CD44 variant expression in follicular cell-derived thyroid cancers, highlighting its potential as both a diagnostic biomarker and therapeutic target [18,60,61,62,63]. These investigations reveal distinct patterns of CD44 variant expression across TC subtypes (Table 1), underscoring their potential applications in improving diagnosis and guiding therapeutic strategies.

### 3.1. Papillary Thyroid Carcinoma

**Figure 3:**
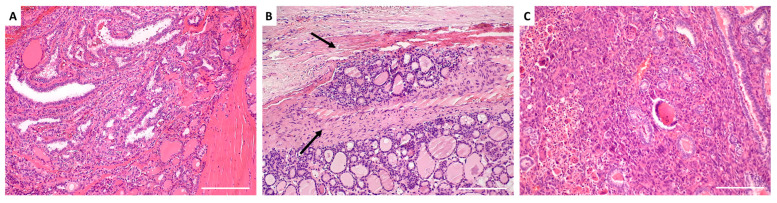
Histological architecture of thyroid carcinoma subtypes. (**A**) Papillary thyroid carcinoma: The tumor exhibits complex papillary structures with central fibrovascular cores, lined by overlapping cells with optically clear nuclear chromatin. (**B**) Follicular thyroid carcinoma: The tumor is characterized by compact, well-formed follicles lined by hyperchromatic follicular epithelial cells. Intravascular invasion within an extrathyroidal capsular blood vessel is evident (arrows). (**C**) Anaplastic thyroid carcinoma: The tumor appears solid and consists of a malignant mixture of pleomorphic cells and multinucleated giant cells. Background PTC is visible on the right. Images were captured at the Department of Anatomical Pathology, University of Pretoria, using an Olympus light microscope at 4× magnification with a 5500 µm scale bar.

PTC, the most common well-differentiated subtype of TC, exhibits significant gross variability and multiple histopathologic variations. Its microscopic hallmarks include papillae with fibrovascular cores lined by single or multiple layers of cuboidal cells, while the characteristic nuclear features—optically clear chromatin (ground glass appearance), intranuclear cytoplasmic inclusions, and intranuclear grooves—are sufficient for diagnosis even in the absence of fibrovascular cores (Figure 3A) [68]. The aggressive subtypes—columnar cell, diffuse sclerosing, tall cell, and solid variants—are linked to higher rates of multifocality, extrathyroidal extension, metastasis, and recurrence [69]. Studies have shown that PTC exhibits an increase in CD44 mRNA isoforms, distinguishing it from histologically normal thyroid tissues, and that most PTCs overexpress CD44 in contrast to normal follicular cells [70,71,72].

A study by Figge et al. demonstrated that 97% of PTC cases exhibited strong CD44 expression, with an intense plasma membrane staining pattern using the A3D8 anti-human CD44 monoclonal antibody (mAb) [73]. Further characterization using the 2F10 anti-human CD44v6 mAb, a marker associated with tumor metastasis, revealed positivity in all 11 tested cases of PTC. These findings suggest that deregulated CD44v6 expression in PTC contributes to lymph node metastasis and may allow tumor cells to remain dormant for extended periods [73]. Similarly, Ermak et al. demonstrated that PTC exhibits specific patterns of CD44 variants, including exons v6, v7, v8, v9, and v10, with weak expression of v3. Notably, several isoforms detected in PTC were absent in histologically normal thyroid tissue from the corresponding contralateral lobes, suggesting that these aberrant splicing patterns could serve as distinguishing markers for PTC [70].

Takano et al. analyzed CD44 variant expression in thyroid tumors using reverse transcription-polymerase chain reaction (RT-PCR), and their findings revealed increased expression of CD44 variants in most FTC, particularly in follicular carcinomas, poorly differentiated PTC, and some FAs. However, these variants were scarcely detectable in ATC, indicating that CD44 variant expression may be associated with the proliferation of differentiated thyroid cells [74].

Overexpression of CD44v6 has been associated with lymph node metastasis and recurrence in PTC. Co-expression of CD44v6 and MMP2 correlates with tumor progression and metastasis [64,73,75]. MMP-2 is a proteolytic enzyme that degrades extracellular matrix components, facilitating tumor cell invasion and migration. The positive correlation between CD44v6 and MMP-2 protein levels in PTC specimens underscores the synergistic role of these molecules in enhancing metastatic potential. This highlights CD44v6 as not only a diagnostic and prognostic marker but also a potential therapeutic target for reducing metastatic risk. In addition, an increased CD44v8-10/CD44s ratio has been linked to aggressive disease phenotypes in PTC [12].

### 3.2. Follicular Thyroid Carcinoma

Most FTC lesions are slow-growing, painless, cold on scintigraphy, and exhibit less phenotypic heterogeneity than PTC [76]. FTC is distinguished from FA by capsular or vascular invasion (Figure 3B), with extensive invasion linked to higher metastatic potential and mortality [68]. FTC commonly spreads hematogenously, with metastases occurring in the bone, liver, lungs, and other distant sites. Regional lymph node metastases are very rare [77].

To explore the expression of CD44 in FTC, Figge et al. reported that 56% (9 of 16 cases) of follicular adenomas (FAs) exhibited CD44 expression with plasma membrane staining. In contrast, FTC showed CD44 expression in 38% (three of eight cases), and Hurthle cell neoplasms in 50% of cases (four of eight) [73]. These early findings hinted at the differential expression of CD44 between benign and malignant thyroid lesions, setting the stage for further investigation into its diagnostic and prognostic relevance. In 1996, Takano et al. observed increased that CD44 variant expression was increased in most follicular tumors compared with normal thyroid tissues, particularly in FTCs, as well as some FAs [74]. Notably, CD44 splice variants were more highly expressed in FTC than in FA. This differential expression helped to distinguish malignant from benign thyroid lesions and pointed to the potential of CD44 variants as biomarkers for FTC diagnosis [18,63].

Further supporting this, Gasbarri et al. examined 157 thyroid specimens, including normal, benign, and malignant lesions, and found that normal thyrocytes did not express CD44v6. In contrast, CD44v6 expression varied among benign and malignant lesions, with increased levels associated with malignant transformation. Their findings suggested that the simultaneous expression of CD44v6 and galectin-3 enhances the diagnostic differentiation between benign and malignant thyroid nodules, highlighting a potential diagnostic application [78].

### 3.3. Anaplastic Thyroid Carcinoma

ATC is a highly aggressive tumor with a nearly 100% disease-specific mortality rate [69]. This cancer subtype often coexists with other forms of TC and may arise from well-differentiated TC, especially PTC and FTC [79]. Due to its extreme heterogeneity, ATC displays a variety of differentiated cells, including pleomorphic giant, epithelioid, and spindle cells, making it a particularly challenging malignancy to diagnose and treat (Figure 3C) [80].

In terms of CD44 expression, Takano et al. reported that CD44 variants were barely detectable in ATC when assessed using RT-PCR with a fluorescent image analyzer [74]. However, subsequent studies have shown contrasting findings. High expression of CD44 variants has been associated with stem-like properties and chemoresistance in ATC, both of which are linked to the tumor’s aggressive behavior and poor prognosis. These findings underscore the potential role of CD44 variants in driving malignancy in ATC and their involvement in tumor progression, making them valuable biomarkers for aggressive tumor behavior and treatment resistance [81,82].

While these studies provide important insights, there remains a lack of comprehensive and consistent findings regarding the exact role of CD44 variants in ATC. This gap in research limits our ability to definitively determine their diagnostic and therapeutic potential in this highly aggressive tumor. Further studies are necessary to clarify the exact relationship between CD44 variant expression and ATC’s clinical outcomes, which could enhance targeted therapeutic strategies for this challenging cancer.

## 4. Multidrug Resistance in Follicular Cell-Derived Thyroid Cancers

**Figure 4:**
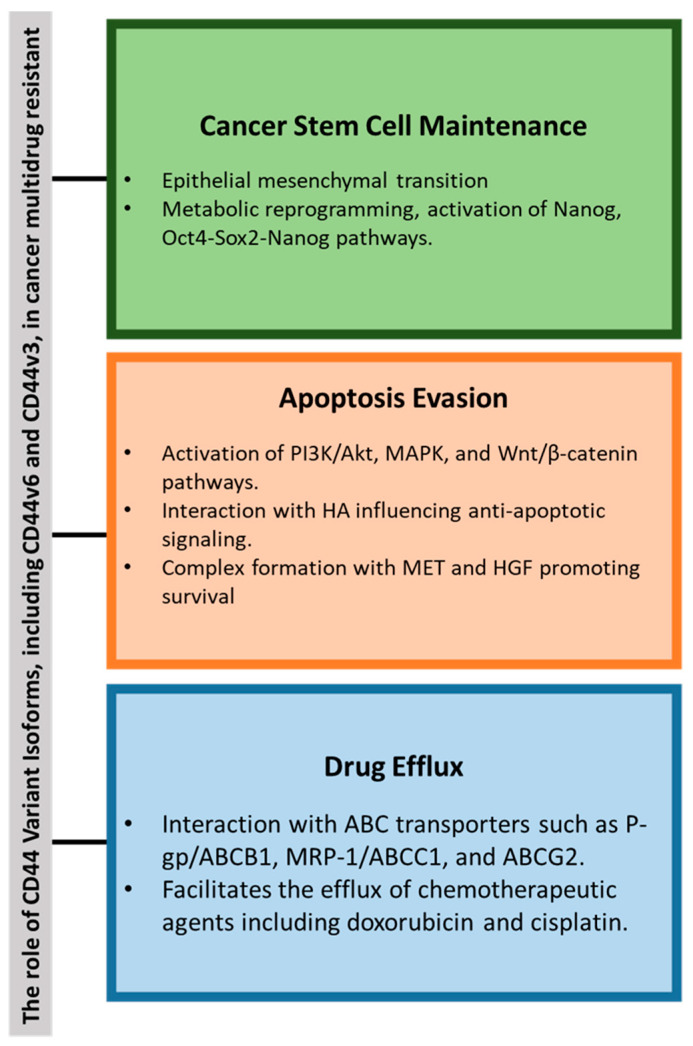
Summary of the key role of CD44 variants in cancer multidrug resistance (MDR) through the following: (1) cancer stem cell (CSC) maintenance via epithelial-mesenchymal transition (EMT) and metabolic reprogramming; (2) evasion of apoptosis through activation of PI3K/Akt, MAPK, and Wnt/β-catenin pathways; and (3) enhanced drug efflux. Abbreviations: ABCB1—ATP-Binding Cassette Subfamily B Member 1, ABCG2—ATP-Binding Cassette Subfamily G Member 2, Akt—Protein Kinase B, CD44—Cluster of Differentiation 44, MAPK—Mitogen-Activated Protein Kinase, OCT4—Octamer-Binding Transcription Factor 4, P-gp—P-Glycoprotein, PI3K—Phosphoinositide 3-Kinase, SOX-2—SRY-Box Transcription Factor 2, Wnt—Wingless/Integrated.

Elevated levels of CD44 expression have been associated with MDR in various cancers, including TCs [60,83,84,85]. CD44 interacts with the extracellular matrix, particularly HA, to activate key signaling pathways such as Wnt/β-catenin and PI3K/Akt, which enhance the expression of drug-resistance genes [86]. CD44 variant isoforms (e.g., CD44v6 and CD44v3) contribute to the pre-metastatic niche, tumor progression, and chemotherapy resistance through pathways involving Nanog, Oct-Sox2-Nanog, and other regulators; see Figure 4 [86,87].

### 4.1. Mechanisms of CD44-Driven MDR

#### 4.1.1. Enhanced Drug Efflux: Interaction with ATP-Binding Cassette Transporters

CD44 promotes MDR by interacting with ABC transporters such as P-glycoprotein (P-gp/ABCB1), MRP-1 (ABCC1), and breast cancer resistance protein (ABCG2). These transporters facilitate the efflux of chemotherapeutic agents, reducing their intracellular concentrations and efficacy [88,89]. In cancers that share common chemotherapy regimens with ATC, MDR mechanisms involve overexpression of ABC transporters, contributing to resistance against commonly used drugs like doxorubicin, cisplatin, and paclitaxel, drugs also utilized in ATC treatment [90,91]. Clinical studies have shown that elevated CD44v8-10 expressions have been associated with cisplatin resistance. Immunohistochemical analysis demonstrated that patients with higher CD44v9 (detecting CD44v8-10) expression had significantly lower 5-year cancer-specific survival rates [92]. Similarly, High CD44v8-10 expression in CSCs has been associated with poor prognosis in esophageal squamous cell carcinoma patients treated with chemoradiotherapy. This suggests that CD44v8-10 contributes to treatment resistance and adverse clinical outcomes [93].

Studies have demonstrated that the inhibition of ABC transporters (with gefitinib or verapamil) can restore chemosensitivity in ATC cell lines [94,95]. Kaplan–Meier survival analyses indicated that high ABCC1 expression is associated with reduced OS in numerous cancer types [96]. The co-expression of multiple ABC transporters, such as ABCB1 and ABCA1, is a key factor in the development of MDR in TC [97]. However, while the role of ABC transporters in other malignancies treated with similar chemotherapy drugs is well-established, their specific contribution to ATC remains insufficiently characterized. This raises a fundamental question: is ATC drug resistance driven by the same ABC transporters identified in other cancers, or does ATC harbor unique ABC transporters that support tumor growth, metastasis, and therapeutic resistance? Addressing this gap is essential for optimizing treatment strategies and overcoming MDR in ATC. Table 2 summarizes the ABC transporters expressed in chemotherapeutic substrates that are commonly utilized by ATC.

#### 4.1.2. Cancer Stem Cell Maintenance: Promotion of Stem-Like Phenotypes

CD44 is a pivotal CSC receptor, linked to metastasis, therapy resistance, and recurrence [105]. Its overexpression reduces chemotherapy efficacy in various cancers [106]. CD44 is a key marker for isolating CSCs, either alone or with others such as CD24, CD133, and CD34 [13]. Research shows that irradiation or EMT upregulates stemness markers, increasing CD24−/low/CD44+ cell populations indicative of CSCs, as observed in breast cancer [107].

Ryu et al. [108] demonstrated that the expression of CSC markers, specifically CD44+ and CD24−, in PTC tissue samples was significantly associated with reduced recurrence-free survival. The co-expression of CD44+ and CD24− was found to exert a pronounced negative impact on recurrence-free survival and was strongly correlated with the presence of gross extra-thyroidal extension [108].

Metabolic reprogramming also maintains CSC phenotypes. Histone deacetylase 11 promotes glycolysis through the liver kinase B1/AMP-activated protein kinase pathway, sustaining stemness in hepatocellular carcinoma [109]. In breast cancer, switching from oxidative phosphorylation to glycolysis fosters the CD44+/CD24low/EPCAM+ basal-like CSC phenotype [110].

CD44 variant isoforms are critical for CSC maintenance and tumor progression. In mouse models, CD44v—but not CD44s—drives tumor initiation [111]. Specific isoforms, such as CD44v6 and CD44v8-10, are associated with aggressive cancers, including colorectal and gastric cancers [112,113], while CD44v3 is linked to CSCs in head and neck cancers [114,115].

Unlike the broadly expressed CD44s, CD44v isoforms are restricted to cancer cells and linked to poor prognosis [13,42]. Isoform switching from CD44s to CD44v during EMT underscores their role in metastasis and stemness acquisition [116,117,118,119].

#### 4.1.3. Apoptosis Evasion: Activation of Anti-Apoptotic Pathways

CD44 plays a crucial role in MDR through the activation of intracellular signaling pathways regulating cell survival and proliferation. The interaction between CD44 and HA activates Rho and subsequently the PI3K/Akt pathway, forming a positive feedback loop that sustains Akt activation, counteracts apoptosis, and promotes cellular survival [120]. CD44 also activates p38 MAPK, enhancing cell proliferation [121], and engages extracellular-regulated kinases-1/2, which regulate endothelial cell proliferation and migration [122].

The CD44v6 isoform forms a trimeric complex with MET and hepatocyte growth factor, activating RAS-MAPK, PI3K/Akt, and MET transcription pathways, contributing to chemoresistance and tumor progression [123]. CD44 also regulates cytoskeletal reorganization via interactions with Merlin and Ezrin, Radixin, and Moesin proteins. Merlin suppresses RAS signaling, while its phosphorylation inactivation enhances CD44’s pro-survival roles [124,125].

Additionally, CD44 modulates the Wnt/β-catenin pathway. In leukemia, CD44 downregulation reduces β-catenin levels, inducing cell cycle arrest and inhibiting proliferation [126,127]. These findings highlight CD44’s pivotal role in MDR through its regulation of anti-apoptotic and proliferative signaling pathways.

## 5. Therapeutic Implications and Strategies to Overcome MDR

**Figure 5:**
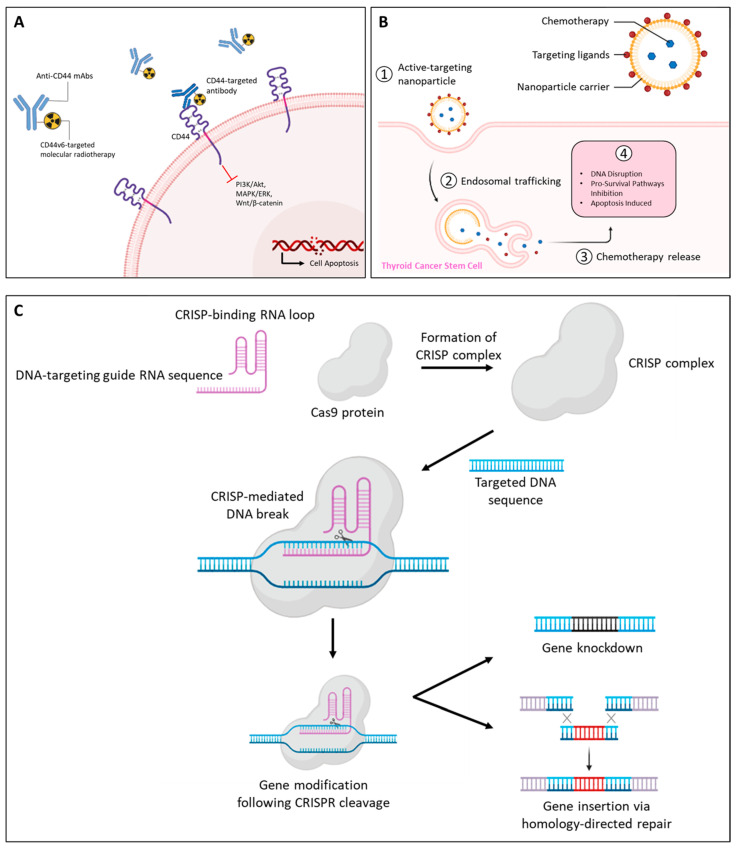
Therapeutic strategies for targeting CD44 in thyroid cancers. Approaches include (**A**) monoclonal antibodies against CD44 isoforms, (**B**) nanoparticle-based drug delivery systems and, (**C**) gene silencing via RNA interference or CRISPR/Cas9 to inhibit cancer progression and metastasis. Abbreviations: CRISPR/Cas9, Clustered Regularly Interspaced Short Palindromic Repeats and CRISPR-associated protein 9; CS, chondroitin sulfate.

MDR poses a significant challenge in cancer treatment, particularly in aggressive TCs. Targeting CSCs, which contribute to tumor persistence and resistance, has emerged as a promising strategy. CD44, a key marker in the CSC niche, has gained considerable attention as both a therapeutic target and a potential biomarker. Strategies to overcome MDR by targeting CD44 include monoclonal antibodies, nanocarrier-based drug delivery systems, and innovative gene-editing technologies; see Figure 5 [128,129,130,131,132].

### 5.1. Targeting CD44 Variants in Thyroid Cancers: A Translational Perspective

While CD44 expression and MDR in TC have been established, targeted therapeutic strategies remain largely unexplored. However, successful interventions in other CD44-driven malignancies—including head and neck squamous cell carcinoma, breast cancer, osteosarcoma, and leukemia [37]—provide a compelling rationale for investigating similar approaches in TC. This section explores three emerging strategies—mAbs, nanoparticle-based drug delivery, and CRISPR/Cas9 gene silencing—which have demonstrated efficacy in overcoming MDR in other cancers and may hold therapeutic potential for TC.

#### 5.1.1. CD44-Targeted Antibodies

Numerous anti-CD44 mAbs, including anti-pan-CD44 mAbs, C_44_Mab-5, and C_44_Mab-46 [133], have been previously developed against CD44s and CD44 variants and applied to antibody-drug conjugates and chimeric antigen receptor T-cell therapy [134] (Figure 5A). They have demonstrated efficacy in cancer models, reducing tumor growth, metastasis, and post-radiation recurrence [135].

Several mAb-derived treatments for solid tumors have been approved, while others are under clinical investigation. One notable example is RG7356, a humanized anti-CD44 IgG1 monoclonal antibody, which has been evaluated in phase I clinical trials for patients with advanced CD44-expressing solid tumors. While the study confirmed its safety, it was terminated early due to the absence of clear clinical or pharmacodynamic dose–response relationships, preventing the establishment of an optimal dosing schedule [133,136,137]. In chronic lymphocytic leukemia, RG7356 induced cytotoxicity in malignant B cells without affecting normal B cells, underscoring its specificity [138]. Targeting CD44v6 has also shown promise. mAb U36 exhibited significant tumor uptake in head and neck squamous cell carcinomas [139], while VFF18, a murine-derived antibody against CD44v6, displayed selective and rapid tumor targeting [140].

Collectively, these findings suggest that CD44 isoform expression, particularly CD44s, can serve as a predictive biomarker for therapy response [141]. Furthermore, CD44v6 expression, often associated with poor prognosis in advanced PTC, FTC, and ATC, presents an opportunity for molecular radiotherapy. Combining CD44v6-targeted radiotherapy with tyrosine kinase inhibitors such as sorafenib, dabrafenib, or trametinib holds the potential for enhanced therapeutic outcomes [61].

#### 5.1.2. Nanoparticle Drug Delivery

Nanotechnology-based drug delivery systems have revolutionized cancer therapy by enhancing precision and reducing off-target toxicity. CD44-targeted nanoparticles, functionalized with HA or chondroitin sulfate (CS), facilitate efficient tumor targeting and cellular internalization. HA-coated nanocarriers, including dendrimers [142], micelles [143], nano-emulsions [144], and nanogels [145], show promise in overcoming MDR mechanisms by bypassing efflux transporters such as P-glycoprotein (Pgp).

These carriers enable controlled drug release, targeted accumulation at tumor sites, and reduced systemic toxicity (Figure 5B). For instance, HA-based liposomes designed to target CD44-positive CSCs have been shown to enhance therapeutic efficacy and inhibit tumor growth [146]. Additionally, nanoparticle-mediated delivery of therapeutic agents, such as doxycycline, has demonstrated significant potential for overcoming MDR in CD44-overexpressing MCF-7 breast cancer cells, offering a promising approach to targeted chemotherapy [142,147].

#### 5.1.3. Gene Silencing Approaches

Advances in gene-editing technologies have opened new avenues for targeting CD44. The Clustered Regularly Interspaced Short Palindromic Repeats and CRISPR-associated protein 9 (CRISPR/Cas9) system, leveraging sequence-specific DNA cleavage, offers precision in downregulating CD44 variants (Figure 5C). Liu et al. reported that silencing CD44 in highly metastatic human osteosarcoma cells using CRISPR/Cas9 inhibited their proliferation, migration, and invasion [148]. These findings highlight the critical role of CD44 in tumor aggressiveness and its potential as a therapeutic target.

RNA interference approaches have also shown promise in silencing CD44 variants, leading to reduced tumor growth and enhanced sensitivity to chemotherapeutics [149,150]. The integration of CRISPR/Cas9 and RNA interference strategies into TC research could provide novel insights and therapeutic options for combating MDR, particularly in tumors expressing CD44 variants.

## 6. Challenges and Limitations in CD44-Targeted Therapies

One of the major challenges in studying and targeting CD44 in follicular cell-derived thyroid cancers is its heterogeneity [80]. Significant intra- and inter-tumoral variability in CD44 expression complicates the identification of consistent patterns across different tumors. Furthermore, notable differences in CD44v expression between primary tumors and their metastatic sites highlight the complexity of the tumor microenvironment, making targeted therapeutic approaches less predictable [59].

CD44 is widely expressed in normal tissues, posing a risk of off-target effects when developing CD44v-specific therapies [28]. The ubiquitous nature of CD44 across various non-malignant cell types increases the likelihood of potential toxicity, raising concerns about the therapeutic window and patient safety in clinical applications.

Translational barriers from preclinical models to clinical trials represent a significant hurdle. The lack of robust and predictive preclinical models can lead to inconsistencies when transitioning to human studies. Additionally, the validation of CD44v as a reliable biomarker remains limited, impacting the ability to stratify patients effectively for targeted therapies. These technical and clinical challenges must be addressed to improve the translational potential of CD44v-based approaches.

## 7. Future Directions and Perspectives

Previous studies have shown a considerable correlation between the expression of CD44, tumor aggressiveness, and MDR [42,86]. However, the expression of CD44 variations in various TC subtypes, especially to their patterns of spatial expression and correlation with clinicopathological characteristics, remains limitedly known. Similarly, there is little information on how these variations affect aggressiveness and chemoresistance in more advanced forms, such as ATC, compared to PTC and FTC.

Using methods like immunohistochemistry [63], RT-PCR [151], and more sophisticated single-cell RNA sequencing techniques, a thorough mapping of CD44 variant profiles across different TC subtypes, stages, and geographic populations could close this gap and offer insightful information. Determining the predictive importance of CD44 in TC would need a comprehensive evaluation of CD44 expression in normal versus malignant thyroid tissues and its association with clinicopathological indicators (such as tumor size, lymph node metastasis, and patient survival). Similar methods might be applied to TC to provide a more thorough understanding of CD44 variant expression across various cell groups inside tumors. A number of recent studies have started investigating the usefulness of single-cell RNA sequencing in mapping tumor heterogeneity.

Further research is needed to fully understand the molecular mechanisms behind CD44-mediated MDR. Tumor growth and treatment resistance are largely controlled by pathways like Wnt/β-catenin, PI3K/Akt, and interactions with ABC transporters. According to recent studies, these pathways can be modulated by CD44 variant isoforms, indicating that TC subtypes may exhibit chemoresistance as a result of CD44-mediated control of important molecular signaling pathways [85,88,89,107]. Particularly in aggressive TC subtypes like ATC, experimental models such as in vitro, in vivo, and organotypic 3D cultures should concentrate on how individual CD44 variations interact with these pathways to increase therapeutic resistance. Finding targetable signaling intermediates within these pathways may open up new treatment options for MDR.

There is great potential for conquering MDR by incorporating CD44 variant profiles into individualized therapy strategies. The possibility of combining clinical data, genetic profiling, and CD44 expression data to maximize customized treatment strategies for TC has been shown by recent developments in predictive modeling. CD44+ cells in ATC exhibited stem-like characteristics, as demonstrated in a study by Kim et al., highlighting the potential of CD44 as a biomarker for therapy-resistant cell populations [62]. Immunophenotyping and genomic profiling of CD44-expressing cells in TC may help with patient stratification and enhance treatment outcome prediction. For high-risk or treatment-resistant instances, this would be especially pertinent, allowing physicians to develop more potent combination medicines, such as immunotherapy and targeted therapies.

MDR is influenced by the TME, with CD44 playing a pivotal role in these interactions. Recent studies suggest that CD44 facilitates crosstalk between tumor cells and key components of the TME, including immune cells, HA, and matrix MMPs. These interactions contribute to tumor progression and resistance to therapy by modulating extracellular matrix remodeling, immune evasion, and cell adhesion dynamics [28,107,152]. Future studies should focus on understanding how CD44 variations influence these interactions in TC, given the critical role of the TME in chemoresistance. HA–CD44 interactions have been shown to regulate tumor cell adherence and motility, potentially increasing chemotherapy resistance. To improve treatment outcomes and prevent resistance in TC, therapeutic strategies targeting CD44 could be combined with immunotherapies or anti-MMP drugs. Early-phase clinical trials are necessary to evaluate the efficacy of CD44-targeting treatments.

Multicenter clinical trials and prospective investigations are necessary to validate the diagnostic, prognostic, and therapeutic implications of CD44 variations in MDR in order to guarantee clinical translation. Such initiatives would be aided by the biobanking of thyroid cancer tissue samples from various patient groups, which would make it possible to identify the CD44 expression profiles most indicative of treatment resistance. Additionally, cooperation between biologists, bioinformaticians, and oncologists may facilitate data integration and the development of thorough models of the mechanisms underlying CD44-associated resistance in TC.

## 8. Conclusions

CD44 and its variant isoforms play a significant role in cancer progression and MDR across various cancer types. Although direct evidence linking CD44 to TC remains limited, the protein’s well-established functions in other malignancies justify further exploration of its potential role in thyroid tumorigenesis, resistance to therapy, and clinical outcomes. Variations such as CD44v6 and CD44v8-10 are associated with aggressive cancer characteristics, including poor prognosis and treatment resistance, suggesting that similar mechanisms may drive malignancy and MDR in TC [12,64,67,85,153].

CD44 variations could serve as more reliable biomarkers for advanced or chemo-resistant TC compared to conventional markers like thyroglobulin, as they reflect the tumor’s ability to evade apoptosis, maintain stem-like features, and efflux drugs, factors central to MDR [154]. Targeting these variations might enable more effective treatments that circumvent these processes, reducing the risk of misdiagnosis or ineffective therapy in advanced stages of TC.

Various methods, including quantitative PCR, next-generation sequencing, and immunohistochemistry, can assess the relationship between CD44 variations and TC [12,62]. Furthermore, functional assays may help clarify the role of CD44 variations in tumor aggression and therapy resistance. Ultimately, targeting CD44 variations using monoclonal antibodies, nanotechnology-based drug delivery, or gene-editing techniques could enhance therapeutic efficacy, especially in resistant TC cases.

## Figures and Tables

**Table 1:** The expression of CD44 variants in different follicular cell-derived thyroid cancer subtypes.

FCDTC Subtypes	CD44 Variant	Function	Reference
PTC	CD44v8-10	Reduce cell proliferation	[12]
	CD44v6	Metastasis and lymphatic invasion	[64]
			[63]
FTC	CD44v6	Tumor progression	[65]
ATC	CD44v6	Increased tumor invasiveness and metastatic potential	[66]
	CD44v8-10	Not well-established	[67]

Abbreviations: ATP—Anaplastic thyroid carcinoma, FTC—Follicular thyroid carcinoma, PTC—Papillary thyroid carcinoma.

**Table 2:** ABC transporters expressed in anaplastic thyroid cancer (ATC) and their associated chemotherapeutic substrates. Due to limited data, the listed chemotherapeutic substrates for each transporter are based on findings from various cancer studies, not exclusively ATC research. Only commonly used ATC chemotherapy drugs are included. Adapted from [98].

ATC Chemotherapeutic Drug	ABC Transporter(s)
Carboplatin	ABCA8 [99]
Cisplatin	ABCB10 (MTABC2) [100,101] ABCG2 (BCRP) [102]
Docetaxel	ABCB1 (MDR1, P-gp) [103] ABCC10 (MRP7) [99]
Doxorubicin	ABCB1 (MDR1, P-gp) [99,103] ABCB10 (MTABC2) [99] ABCC1 (MRP1) [99,104] ABCC10 (MRP7) [99] ABCC5 (MRP5) [99] ABCG1(ABC8) [99] ABCG2 (BCRP) [94,99,102]

Abbreviations: ABCC10—ATP-Binding Cassette Subfamily C Member 10, ABCG1—ATP-Binding Cassette Subfamily G Member 1, ABCG2—ATP-Binding Cassette Subfamily G Member 2, ABC8—ATP-Binding Cassette 8, BCRP—Breast Cancer Resistance Protein, MDR1—Multidrug Resistance Protein 1, MRP1—Multidrug Resistance-Associated Protein 1, MRP5—Multidrug Resistance-Associated Protein 5, MRP7—Multidrug Resistance-Associated Protein 7, MTABC2—Mitochondrial ATP-Binding Cassette Transporter 2, P-gp—P-Glycoprotein.

## Abbreviations

The following abbreviations are used in this manuscript:

ABCA8	ATP-Binding Cassette Subfamily A Member 8
ABCB1	ATP-Binding Cassette Subfamily B Member 1
ABCB10	ATP-Binding Cassette Subfamily B Member 10
ABCG1	ATP-Binding Cassette Subfamily G Member 1
ABCG2	ATP-Binding Cassette Subfamily G Member 2
ABCG8	ATP-Binding Cassette Subfamily G Member 8
ABCC1	ATP-Binding Cassette Subfamily C Member 1
ABCC10	ATP-Binding Cassette Subfamily C Member 10
ABCC5	ATP-Binding Cassette Subfamily C Member 5
ABCC7	ATP-Binding Cassette Subfamily C Member 7
ABCF2	ATP-Binding Cassette Subfamily F Member 2
Akt	Protein Kinase B
ATC	Anaplastic Thyroid Carcinoma
β-catenin	Beta-Catenin
CD44	Cluster of Differentiation 44
CD44v	CD44 Variant
CSC	Cancer Stem Cell
ECM	Extracellular Matrix
EMT	Epithelial-Mesenchymal Transition
FA	Follicular Adenoma
FTC	Follicular Thyroid Carcinoma
HA	Hyaluronic Acid
MAPK	Mitogen-Activated Protein Kinase
mAb	Monoclonal Antibody
MDR	Multidrug Resistance
MET	Mesenchymal-Epithelial Transition Factor
MMP	Matrix Metalloproteinase
mRNA	Messenger RNA
OCT4	Octamer-Binding Transcription Factor 4
PDTC	Poorly Differentiated Thyroid Carcinoma
P-gp	P-Glycoprotein
PI3K	Phosphoinositide 3-Kinase
PTC	Papillary Thyroid Carcinoma
RAS	Rat Sarcoma
RT-PCR	Reverse Transcription-Polymerase Chain Reaction
SOX-2	SRY-Box Transcription Factor 2
TC	Thyroid Cancer
Wnt	Wingless/Integrated
